# Spatiotemporal modeling of COVID-19 spread: unveiling socioeconomic disparities and patterns, across social classes in the urban population of Kermanshah, Iran

**DOI:** 10.3389/fpubh.2024.1400629

**Published:** 2024-10-04

**Authors:** Alireza Zangeneh, Nasim Hamidipour, Zahra Khazir, Arash Ziapour, Homa Molavi, Zeinab Gholami Kiaee, Raziyeh Teimouri, Ebrahim Shakiba, Moslem Soofi, Fatemeh Khosravi Shadmani

**Affiliations:** ^1^Social Development and Health Promotion Research Center, Health Institute, Kermanshah University of Medical Sciences, Kermanshah, Iran; ^2^School of Nursing and Midwifery, Dezful University of Medical Sciences, Dezful, Iran; ^3^Department of Public Health, Torbat Jam Faculty of Medical Sciences, Torbat Jam, Iran; ^4^Cardiovascular Research Center, Imam-Ali Hospital, Kermanshah University of Medical Sciences, Kermanshah, Iran; ^5^Department of Engineering Management, School of Engineering, The University of Manchester, Manchester, United Kingdom; ^6^Department of Statistics, School of Mathematical Science, AmirKabir University of Technology, Tehran, Iran; ^7^UniSA Creative, University of South Australia, Adelaide, SA, Australia; ^8^Research Center for Environmental Determinants of Health (RCEDH), Health Institute, Kermanshah University of Medical Sciences, Kermanshah, Iran

**Keywords:** COVID-19, crisis response strategies, socioeconomic status, GIS, disease transmission, epidemic management

## Abstract

**Background:**

Presenting ongoing outbreaks and the potential for their spread to nearby neighborhoods and social classes may offer a deeper understanding, enable a more efficient reaction to outbreaks, and enable a comprehensive understanding of intricate details for strategic response planning. Hence, this study explored the spatiotemporal spread of COVID-19 outbreaks and prioritization of the risk areas among social classes in the Kermanshah metropolis.

**Methods:**

**I**n this cross-sectional study, the data of 58.951 COVID-19-infected patients were analyzed. In 2020, out of 24.849 infected patients, 10.423 were females, 14,426 were males, and in 2021, 15.714 were females, and 18,388 were males. To categorize social classes (working, middle, and upper), we utilized economic, social, cultural, and physical indicators. Our analysis utilized Arc/GIS 10.6 software along with statistical tests, including standard distance (SD), mean center (MC), standard deviational ellipse (SDE), and Moran’s *I*.

**Results:**

The results revealed that the average epicenter of the disease shifted from the city center in 2020–2021 to the eastern part of the city in 2021. The results related to the SD of the disease showed that more than 70% of the patients were concentrated in this area of the city. The SD of COVID-19 in 2020 compared to 2021 also indicated an increased spread throughout the city. Moran’s I test and the hotspot test results showed the emergence of a clustered pattern of the disease. In the Kermanshah metropolis, 58,951 COVID-19 cases were recorded, with 55.76% males and 44.24% females. Social class distribution showed 28.86% upper class, 55.95% middle class, and 15.19% working class. A higher disease prevalence among both males and females in the upper class compared to others.

**Discussion:**

Our study designed a spatiotemporal disease spread model, specifically tailored for a densely populated urban area. This model allows for the observation of how COVID-19 propagates both spatially and temporally, offering a deeper understanding of outbreak dynamics in different neighborhoods and social classes of the city.

## 1 Introduction

The COVID-19 pandemic is considered the most notable global crisis (1). The highly contagious nature of the disease, often with asymptomatic cases, poses a serious obstacle to the healthcare system (2). Infections can propagate through human contacts within households, communities, or randomly among the general population (3). The WHO declared COVID-19 a public health emergency of international concern on January 30, 2020, leading to its prompt recognition. Subsequently, on March 11, 2020, it was declared that COVID-19 had transitioned into a pandemic (4). Iranian governmental departments confirmed the initial case of a positive SARS-CoV-2 infection on February 19, 2020. The SARS-CoV-2 virus has impacted more than 7.6 million individuals in Iran, resulting in over 146,480 reported fatalities as of August 2023 (5).

Detecting the spatial spread of infectious disease outbreaks in a timely manner is crucial for implementing successful control strategies and provides valuable information about important risk variables (6, 7). Infectious diseases seldom follow straightforward patterns (8). COVID-19, as an infectious disease with spatial dimensions, has significantly advanced researchers’ understanding of its epidemiology. Visualizing outbreak dynamics stands out as a prevalent method, enabling the easy identification of spatial patterns and the selection of specific areas for in-depth studies. By uncovering these spatial patterns, researchers can gain better insights into disease dynamics, contributing valuable information for policymaking in managing outbreaks (9). Maps generated through these techniques are particularly effective in pinpointing the origin of an outbreak and showing how it spreads over time. When an epidemic occurs, tracking the spatial and temporal movement of outbreaks through neighbourhoods becomes instrumental in pinpointing origins and trends. The Geography Information System (GIS) is used to understand, analyze, intervene, and display different maps, focal positions, and big data, particularly in the realm of health development. GIS technology helps establish the geographical characteristics of people’s living places, develop evidence-based policies, and observe interventions (10). This makes GIS a crucial tool for quickly reviewing information about epidemics, understanding their development patterns, and offering timely support for decisions and preventive measures (11). However, there is a gap in the literature, as indicated by references (12, 13) due to the absence of studies in Iran that specifically concentrate on employing GIS to investigate the spatial distribution of diseases among distinct social classes.

Previous research indicates that the adverse impacts of the pandemic on economic conditions, hospitalization, mental health, and subjective well-being vary among people (14, 15). Studies highlight disparities in subjective well-being and employment stability based on individuals’ employment situations, with these differences intensifying during the pandemic (16, 17). Gender imbalances are reinforced as females are more likely to experience job loss and reduced earnings, exhibiting lower job performance than males (18). Education level influences individuals’ responses to social policies, with lower-educated individuals responding more vigorously (19).

COVID-19 also has implications for children’s well-being, impacting them through variations in support from their households (20, 21). While there are exceptions, socially advantaged individuals (such as men) may be more adversely affected (22), contributing to the exacerbation of pre-existing social imbalances (23). The pandemic not only intensifies existing social inequalities but also creates new social distinctions (24). Additionally, there is evidence that COVID-19 risk factors are geographically distributed disproportionately, showing a tendency for cluster patterns in areas defined by rural, racial/ethnic, and socioeconomic aspects (25). Socioeconomic status is considered a relevant factor in both individual illness and the spread of the disease (26). Past epidemics have shown that adverse health outcomes are more prevalent in low socioeconomic status groups due to insufficient healthcare access, unfavorable living conditions, and educational barriers (27).

Iran has been one of the most severely affected countries throughout the COVID-19 pandemic. It is a large and complex country with significant disparities in public health and socio-economic conditions, making the spatial spread of the virus across provinces multifaceted and without a singular explanation. Mortality risks tend to be higher for older men, whereas young adults and women face higher risks of infection. The pandemic has been particularly severe in the poorest and most unequal regions of Iran, such as the western provinces. These areas have experienced a high number of infections and elevated mortality risks due to their deprived socioeconomic status and inadequate healthcare conditions (28).

Considering the unique characteristics of the western provinces of Iran, this study analyzes the spatial patterns and driving factors of COVID-19 spread in the city of Kermanshah. We found that previous studies had not focused on the socio-economic factors in Kermanshah. Therefore, we investigated the spatial–temporal trends of the disease within the city and examined how unveiling socioeconomic disparities and patterns across social classes impacts COVID-19 rates. This research makes a significant contribution of our study by introducing an innovative approach to understanding and addressing ongoing COVID-19 outbreaks. By emphasizing the potential spread to nearby neighborhoods and social classes, our study aims to deepen our understanding and facilitate more efficient responses to outbreaks. The novel visualization method proposed in this research focuses on specific neighborhoods and social classes, shedding light on local transmission patterns and the movement of the highly contagious disease in an urban context. A notable contribution of our study lies in the use of priority-based hotspots to categorize the spatial spread of the disease across different neighborhoods and socioeconomic classes. This categorization serves as a valuable tool for supporting intervention programs, particularly in resource-constrained circumstances. The research’s emphasis on strategic response planning and its nuanced approach to spatial spread offer practical insights for halting the disease’s transmission, making a meaningful contribution of our study to the field of epidemic response and management.

## 2 Materials and methods

### 2.1 Study design and data collection

This cross-sectional study focused on the social classes within Kermanshah metropolis. The statistical population under study consisted of residents within the Kermanshah metropolis who had contracted COVID-19. Information pertaining to these individuals was obtained from the Kermanshah University of Medical Sciences using the full enumeration method, relying on the infectious disease surveillance system. The data of 58,951 COVID-19-infected patients were analyzed. The data of 58.951 COVID-19-infected patients were analyzed [32.874 males (55.76%) and 26.077 females (44.24%)] in 2020–2021. In 2020, out of 24.849 infected patients, 10.423 (41.95%) were females, 14,426 (58.05%) were males, and in 2021, 15.714 (46.08%) were females, and 18,388 (53.92%) were males. Our study utilized data from the COVID-19 case registration system in the city of Kermanshah. This data included information on individuals who had presented to diagnostic and treatment centers with symptoms related to COVID-19. At these centers, an RT-PCR test, the standard method for COVID-19 diagnosis, was performed on each presenting individual to confirm their infection. Additionally, all demographic and clinical information of each individual was recorded. Individuals with a positive RT-PCR test result were included in the present study. The researchers then analyzed and examined the information of these individuals to derive important findings and insights regarding the characteristics of COVID-19 patients in the city of Kermanshah. The recorded patient information was digitized using the ArcGIS 10.6 software environment. Centers and spatial patterns within the social classes of the city were determined through statistical tests. To categorize social classes (working, middle, and upper), we utilized economic, social, cultural, and physical indicators. It’s worth noting that these indicators have been consistently employed in other studies to measure socioeconomic status in the Kermanshah metropolis (10, 29, 30). The statistical tests used included SD, MC, SDE, and Moran’s *I*.

### 2.2 MC

“The MC is the x and y coordinates of all the characteristics of the study area. We used it to find out the distribution changes or to compare the distribution of the COVID-19 disease according to the following equation:

$X=\overset{N}{\underset{i=1}{\sum }}\frac{\mathrm{Xi}}{N},Y=\overset{N}{\underset{i=1}{\sum }}\frac{Yi}{N}$

Where Xi and Yiare the coordinates of features and N is the total number of features” (11, 31).

### 2.3 SD

It shows the dispersion of the COVID-19 disease around the mean, which is obtained from the following equation (11, 31):

$SD=\sqrt{\frac{\overset{n}{\underset{i}{\sum }}(x_{i}-\bar{X{)}^{2}}}{n}+\frac{\overset{n}{\underset{i}{\sum }}(y_{i}-\bar{Y{)}^{2}}}{n}}$

### 2.4 SDE

“The SDE is a suitable tool for displaying the dispersion levels of a set of points and is calculated as follows (11, 31):

$SDE=\left(\begin{array}{l} var\left(x\right)cov\left(x,y\right)\\ cov\left(y,x\right)var\left(y\right) \end{array}\right)=\frac{1}{n}\left(\begin{array}{l} \overset{n}{\underset{i=1}{\sum }}{\bar{x}}_{i}2_{i}\overset{n}{\underset{i=1}{\sum }}{\bar{x}}_{i}\bar{y}\\ \overset{n}{\underset{i=1}{\sum }}{\bar{x}}_{i}{\bar{y}}_{i}\overset{n}{\underset{i=1}{\sum }}{\bar{y}}_{i}^{2} \end{array}\right)$

where

$\begin{array}{l} var\left(x\right)=\frac{1}{n}\overset{n}{\underset{i=1}{\sum }}{\left(x_{i}-\overset{x}{x}\right)}^{2}=\frac{1}{n}\overset{n}{\underset{i=1}{\sum }}{\bar{x}}_{i}^{2}\\ cov\left(x,y\right)=\frac{1}{n}\overset{n}{\underset{i=1}{\sum }}\left(x_{i}-\bar{x}\right)\left(y_{i}-\bar{y}\right)=\frac{1}{n}\overset{n}{\underset{i=1}{\sum }}{\bar{x}}_{i}\bar{y}i\\ var\left(y\right)=\frac{1}{n}\overset{n}{\underset{i=1}{\sum }}{\left(y_{i}-\bar{y}\right)}^{2}=\frac{1}{2}\overset{n}{\underset{i=1}{\sum }}{\bar{y}}_{i}^{2} \end{array}$

Where $x_{i}$ and $y_{i}$ are the features coordinates, $\bar{x}$ and $\bar{y}$ are the meancenters, and *n* is equal to the total number of features. The rotation angle is calculated through the following equation” (13, 29). Where $x_{i}$ and $y_{i}$ are the deviations of the features from the MC. “The standard deviation of the rotation angle is also calculated through the following equation (11, 31):

σ1,2(∑i=1nx¯i2+∑i=1ny¯i2±∑i=1nx¯i2−∑i=1ny¯i2)2+4(∑i=1nx¯y)2¯2n12

### 2.5 Moran’s *I*

“Moran’s spatial autocorrelation analysis tool examines spatial autocorrelation based on the location of two characteristic values of geographic features. This tool shows that the distribution pattern of these features, considering the value of the characteristic under study, has a cluster or scattered pattern. This analysis evaluates the distribution pattern of features in space by simultaneously considering the location and the feature. The results of this analysis show whether features are randomly scattered or clustered in space” (13, 29). Moran’s *I* is obtained from the following equation (11, 31):

$\begin{array}{l} I=\frac{n}{S_{o}}\frac{\overset{n}{\underset{i=1}{\sum }}\overset{n}{\underset{j=1}{\sum }}w_{j,i}z_{i}z_{j}}{\underset{i=1}{\sum }z_{i}^{2}}\\ S_{o}=\overset{n}{\underset{i=1}{\sum }}\overset{n}{\underset{j=1}{\sum }}w_{i,j}\\ ZI=\frac{I-E\left[I\right]}{\sqrt{V\left[I\right]}} \end{array}$

where

$\begin{array}{l} E\left[I\right]=-1/\left(n-1\right)\\ V\left[I\right]=E\left[I^{2}\right]-E{\left[I\right]}^{2} \end{array}$

Where $z_{i}$ is showing the deviation of the feature distribution from the mean $\left(x_{i}-\bar{X}\right)$, $w_{i}$, j indicated spatial weight between the features *i* and *j*, *n* is equal to the features total number, and $s_{0}$ is the weights sum (11, 31).

## 3 Results

**Figure 1:**
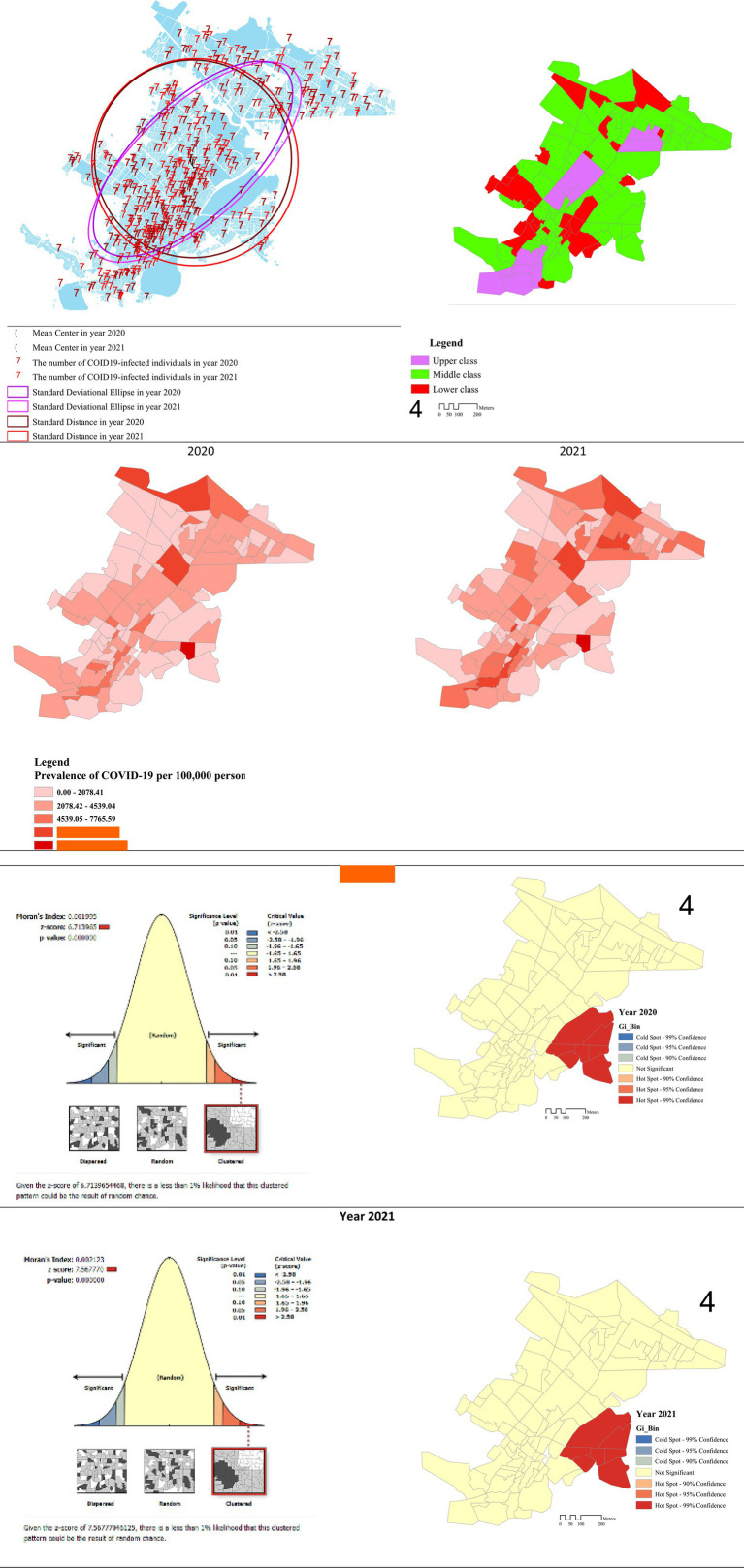
Spatial pattern of the COVID-19 disease in Kermanshah metropolis among the social classes based on tests of SD, MC, SDE, and Moran’s *I*.

Our study indicated that the MC of the disease shifted from the city center between 2020 and 2021, relocating to the eastern part of the city in 2021. Notably, over 70% of the patients were concentrated in this specific area, as illustrated in Figure 1. The results regarding the SD spread of the disease throughout the city, particularly when comparing 2020 to 2021.

The SDE of the disease also exhibited a northeast-southwest direction, and a comparison over the studied years demonstrated an increase in both the height and a change in direction of the ellipse in 2021 (Figure 1). Moran’s *I* spatial autocorrelation and the hotspot test were employed to examine the spatial pattern of COVID-19. The results unveiled the formation of a clustered pattern of the disease throughout the studied years. Specifically, in 2020, the Moran’s I value was 0.001, and it increased to 0.002 in 2021, indicating a higher prevalence of the disease in localized clusters. This suggests that the distribution pattern of COVID-19 in the Kermanshah metropolis has exhibited clustering since the onset of the disease, with the intensity of clustering increasing over time (Figure 1).

The Global Moran’s I results are presented in the left column figures. Figure 1 illustrates relatively similar clusters of COVID-19 in 2020–2021. In both years, Moran’s I values were greater than zero (0.001995, 0.002123) with high significance levels (*p*-values: 0.001) and corresponding z-scores of 6.713965 and 7.567770 for the respective periods mentioned. These findings indicate a significant spatial clustering of COVID-19 cases, emphasizing the consistency and statistical significance of the observed patterns across both years. The results of the hotspot test are presented in the right column figures. The Hotspot analysis classifies polygons into seven distinct categories: three cold spots (indicated by a negative *z*-score) at confidence levels of 99, 95, and 90%, as well as three hotspots (indicated by a positive *z*-score) at the same confidence levels. Lastly, there is a category where the differences are not statistically significant. Each of these seven categories is represented using distinct color schemes, providing a comprehensive visualization of the spatial distribution of COVID-19 clusters and non-significant areas.

In this study, 58,951 people in the Kermanshah metropolis were infected with COVID-19, of which 32,874 (55.76%) were males and 26,077 (44.24%) were females. 28.86% were in the upper class, 55.95% were in the middle class, and 15.19% were in the working class (Table 1).

**Table 1:** Number of COVID-19-infected people, categorized by gender in age and gender groups among the social classes of the Kermanshah metropolis in 2020–2021.

Index	Year	Variables	Upper Class		Middle Class		Working class		Total	
		–	*n*	%	*n*	%	*n*	%	*n*	%
Infected people	2020	Female	3,217	42.89	5,757	41.16	1,449	43.09	10,423	41.95
		Male	4,283	57.11	8,229	58.84	1,914	56.91	14,426	58.05
		Total	7,500	100	13,986	100	3,363	100	24,849	100
	2021	Female	4,483	46.95	8,677	45.79	2,554	45.58	15,714	46.08
		Male	5,065	53.05	10,274	54.21	3,049	54.42	18,388	53.92
		Total	9,548	100	18,951	100	5,603	100	34,102	100
Age group	2020	0–14	132	1.76	246	1.76	81	2.41	459	1.85
		15–64	6,420	85.6	11,885	84.98	2,681	79.72	20,986	84.45
		+65	948	12.64	1,855	13.26	601	17.87	3,404	13.70
	2021	0–14	170	1.78	565	2.98	240	4.28	975	2.86
		15–64	7,892	82.66	15,657	82.62	4,440	79.24	27,989	82.07
		+65	1,486	15.56	2,729	14.40	923	16.48	5,138	15.07
Prevalence per 100,000 population in age groups	2020	0–14	416.6		234.2		150.7		241.0	
		15–64	4413.5		2888.2		3072.6		2817.9	
		+65	24908.0		36230.4		47248.4		46407.6	
	2021	0–14	536.6		538.0		446.2		512.0	
		15–64	5425.4		3804.8		5088.5		3758.3	
		+65	39043.6		53300.7		72562.8		70047.7	
Prevalence per 100,000 population based on gender	2020	Female	3473.9		2214.9		1206.8		2205.5	
		Male	4867.76		3164.5		1570.7		3070.1	
		Total	4153.0		2689.8		1390.1		2636.5	
	2021	Female	4841.0		3338.3		2127.1		3325.1	
		Male	5756.5		3950.9		2505.1		3913.3	
		Total	5287.1		3644.7		2316.0		3618.3	
Total population		Female	922,603		259,916		120,066		472,585	
		Male	87,987		260,037		121,856		469,880	
		Total	180,590		519,953		241,922		942,465	
		0–14	31,680		105,002		53,724		190,406	
		15–64	145,462		411,500		87,255		744,724	
		+65	3,806		5,120		1,272		7,335	

**Figure 2:**
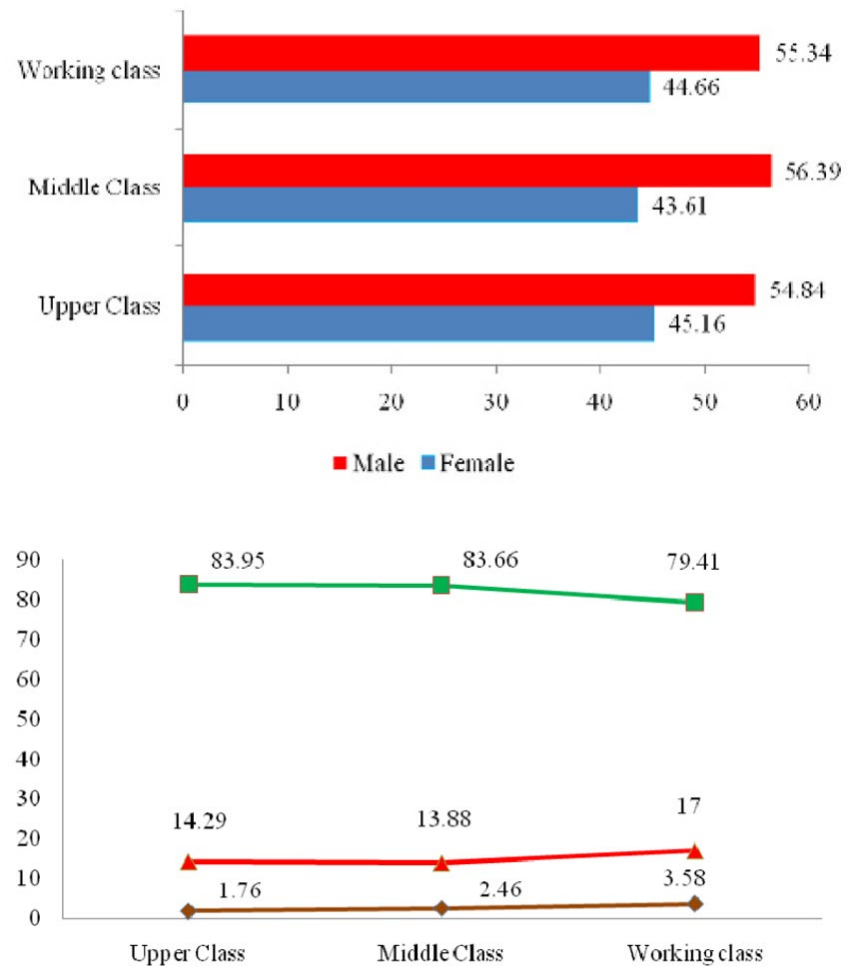
COVID-19 cases categorized by gender and age groups across different social classes in the Kermanshah metropolis.

The results indicated a higher prevalence of COVID-19 in males compared to females. Furthermore, within different social classes, the disease was found to have a higher prevalence among both males and females in the upper class compared to other social classes. The data showed that in 2020, COVID-19 had the highest prevalence among upper-class children, whereas in 2021, the highest prevalence was observed in middle-class children. In the age group of 15–64 years, the highest prevalence of the disease was found in the upper, working, and middle classes, respectively. But in the older adult age group, the working class exhibited the highest prevalence. Overall, the findings demonstrated that the prevalence of COVID-19 in the older adult was higher than in other age groups (Table 2 and Figure 2).

**Table 2:** Number of infected people by COVID-19, by gender in age and gender groups amongthe social classes of the Kermanshah metropolis in 2020–2021.

Variables		Upper Class		Middle Class		Working class		Total	
	–	*n*	%	*n*	%	*n*	%	*n*	%
Infected people	Female	7,683	45.16	14,384	43.61	4,000	44.66	32,874	55.76
	Male	9,330	54.84	18,598	56.39	4,956	55.34	26,077	44.24
	Total	17,013	100	32,982	100	8,956	100	58,951	100
Infected people in age group	0–14	300	1.76	810	2.46	321	3.58	1,429	2.42
	15–64	14,282	83.95	27,593	83.66	7,112	79.41	48,996	83.11
	+65	2,431	14.29	4,579	13.88	1,523	17	8,526	14.46
	Total	17,031	100	32,982	100	8,956	100	58,951	100
Prevalence per 100,000 population in age groups	0–14	946.9		771.4		597.5		750.5	
	15–64	9818.3		7605.4		8150.8		6579.0	
	+65	63872.8		89433.5		119732.7		116237.2	
Prevalence per 100,000 population based on gender	Female	8296.7		5534.1		6398.9		6956.2	
	Male	10603.8		7152.0		7656.5		5549.7	
	Total	9420.7		6343.2		3702.0		6254.9	
Total population	Female	92,603		259,916		120,066		472,585	
	Male	87,987		260,037		121,856		469,880	
	Total	180,590		519,953		241,922		942,465	
	0–14	31,680		105,002		53,724		190,406	
	15–64	145,462		411,500		87,255		744,724	
	+65	3,806		5,120		1,272		7,335	

## 4 Discussion

The COVID-19 pandemic posed a significant threat and caused extensive harm, especially in urban areas. Therefore, this research explores the spatial and temporal spread of COVID-19 outbreaks and identifies high-risk regions across various social strata within the urban area of Kermanshah.

Our study showed that the MC of the disease shifted away from the city center during this study period. Over 70% of the patients were concentrated in a specific area. SD of COVID-19 cases highlighted a significant clustering of patients in this region, indicating a notable spread of the disease throughout the city. The SDE of the disease also exhibited a northeast-southwest orientation, and a comparison over the years studied demonstrated an increase in both the magnitude and a change in orientation of the ellipse. Other studies have shown a strong positive correlation between disease incidence and income inequality as well as median household income in these areas. These socioeconomic disadvantages and inequalities exacerbated during the pandemic; COVID-19 is no exception. As the disease continues to spread, the world has witnessed significant vulnerabilities in healthcare systems, a sharp economic downturn, and rising unemployment rates. For instance, in the United States, those who lose their jobs are at risk of losing their health insurance coverage, further exacerbating existing health and economic disparities (32). This pandemic has created a feedback loop (32, 33).

The results of this study indicated that the COVID-19 disease in the Kermanshah metropolis exhibited a cluster pattern since the onset of the disease, and over time, the intensity of this clustering has increased. Similar results have been observed in other studies, indicating that regardless of its waves, COVID-19 tends to form in clusters (34). Notably, the occurrence of COVID-19 in children showed a high rate in the early stages with a family cluster pattern, with infected children often being asymptomatic carriers and significant contributors to the disease’s spread within communities (35). In these regions, clusters of impoverished communities are marked by reduced median income, limited employment opportunities, and lower educational attainment levels. Areas with lower incomes, a relatively high population density, lower educational attainment, and a higher unemployment rate show a concentration of COVID-19 infections (36). Furthermore, other studies have emphasized that identifying disease clusters is crucial for recognizingat-risk centers that require targeted interventions and other public health measures (37). This underscores the importance of understanding and addressing the clustered nature of COVID-19 for effective public health strategies.

The results indicated a higher prevalence of the disease in males compared to females, aligning with similar findings from previous studies (38, 39). The elevated prevalence in males might be attributed in part to a higher burden of pre-existing diseases and occupational exposure. From an immunological perspective, females may exhibit a more active immune response, potentially offering protection against infectious diseases compared to males. This enhanced immune response is believed to be a result of better responses to pathogens due to higher estrogen levels in females (37, 40). A study conducted in Beijing showed that although COVID-19 had equal prevalence in males and females, infected males, regardless of age, were more vulnerable to experiencing more severe consequences and mortality (39).

A comparison of the disease prevalence among social classes in the Kermanshah metropolis showed that the prevalence in females and males of the upper class was higher than in other social groups. Previous studies have indicatedthat the spread of COVID-19 in wealthier populations may be associated with entertainment activities (41). Therefore, wealthier groups may exhibit more effective results in infection control by limiting recreational activities (34). It is plausible that in the Kermanshah metropolis, the enforcement of quarantine policies and related government restrictions may have provided the upper class with the opportunity for increased engagement in entertainment activities, contributing to the higher disease prevalence in this social class. On the other hand, other studies have stated that “since the poor may have limited access to health facilities, they may remain in the community without treatment or hospitalization, interacting with others, increasing the risk of disease transmission. Another perspective posits that individuals with lower socioeconomic status, often characterized by lower literacy levels, may beless likely to get vaccinated. This population might underestimate the positive effects of vaccination or overestimate the risks associated with it, potentially contributing to a higher prevalence of the disease in the community (42). Studies have shown that socioeconomically disadvantaged people are not necessarily susceptible to the disease, but when they are infected, their communities are at greater risk of widespread disease transmission (34).

Our research findings clarify that the prevalence of the disease in the older adult of the working class was higher than that of other social classes. The older population has been identified in numerous studies as a significantly significant component in the spread of COVID-19 (43, 44). Mansour et al. (2021) showed that a statistically significant correlation between the incidence of COVID-19 in Oman and the population over 65 (44). Moreover, poverty and an aging population were the contributing factors to the COVID-19 pandemic’s spread throughout European countries. Monitoring these significant variables can improve the condition and reduce disease incidence (45). Friesen et al. (2020) demonstrated that the poor are vulnerable to infectious diseases. Their study highlighted that factors such as high building density, crowded living conditions, and poor health contributed to the limited implementation of health measures and policies in impoverished neighborhoods (46). Research has also shown that socioeconomic variables, particularly poverty, could significantly increase the prevalence of COVID-19 (36). Due to limited financial resources, individuals in poverty may face barriers in accessing healthcare facilities, potentially remaining untreated or hospitalized and continuing to interact within the community, thereby increasing the risk of disease transmission. Another hypothesis suggests that lower education levels among impoverished populations may result in lower vaccination rates, with individuals underestimating the benefits or overestimating the risks associated with vaccines, thereby exacerbating the spread of the disease within the community (33).

The findings indicate that in 2020, the disease had the highest prevalence among upper-class children, while in 2021, middle-class children exhibited the highest prevalence. Consistent with other studies, COVID-19 prevalence in affluent communities tended to be higher among children. However, it’s crucial to note that children can present a high prevalence of risk factors for severe lower respiratory tract infections. Shifting resources away from child healthcare to address epidemics among adults may exacerbate the impact on childcare. The results highlight worrisome indirect effects of the epidemic on children’s health, including increased poverty, disruptions in education, inadequate access to school feeding programs, interruptions in vaccination, and reduced access to healthcare facilities and other children’s health programs.

### 4.1 Limitations

This study comes with certain limitations. Firstly, its reliance on cross-sectional data prevented the examination of the long-term spatial pattern of COVID-19-infected individuals. Secondly, our study did not delve into the influence of environmental, behavioral, and genetic factors on COVID-19, which are crucial contributors to the dynamics of the disease. Future research is recommended to address these limitations and explore the impact of these factors for a more comprehensive understanding of the complexities associated with COVID-19.

## 5 Conclusion

Spatial techniques are valuable for identifying various distribution patterns and hotspots of diseases, as well as detecting significant risk factors. COVID-19 manifested with a pronounced clustering trend in urban Kermanshah from the onset of the outbreak, with clusters intensifying over time. Substantial disparities in COVID-19 prevalence among different social classes were observed throughout Kermanshah during the pandemic. Therefore, enhancing access to electronic healthcare data and spatial analysis techniques holds considerable potential for policymaking. These findings can guide policymakers in implementing necessary measures to combat infectious diseases across different social strata.

## Data Availability

The original contributions presented in the study are included in the article/supplementary materials, further inquiries can be directed to the corresponding author.
